# Small bowel volvulus with chylous ascites: a case report

**DOI:** 10.1093/jscr/rjae017

**Published:** 2024-01-30

**Authors:** Shun Nakamura, Masaaki Tajima, Naoki Yokoyama, Nobuyuki Kikuchi

**Affiliations:** Department of Surgery, Shinbeppu Hospital, 3898 Oaza Tsurumi, Beppu 874-0833, Oita, Japan; Department of Surgery, Shinbeppu Hospital, 3898 Oaza Tsurumi, Beppu 874-0833, Oita, Japan; Department of Surgery, Shinbeppu Hospital, 3898 Oaza Tsurumi, Beppu 874-0833, Oita, Japan; Department of Surgery, Shinbeppu Hospital, 3898 Oaza Tsurumi, Beppu 874-0833, Oita, Japan

**Keywords:** small bowel volvulus, chylous ascites, adults

## Abstract

Here, we report a rare case of small bowel volvulus with chylous ascites. A 93-year-old man with a medical history of angina pectoris presented to the emergency department with abdominal pain. Computed tomography revealed a whirl sign of the mesenteric vessels with the axis of the superior mesenteric artery. A diagnosis of small bowel volvulus was made, and emergency surgery was performed. Laparoscopic examination revealed chylous ascites. Due to severe intestinal edema and difficulty in manipulating the forceps, surgery was transferred to a laparotomy. The entire small bowel was twisted 360° counterclockwise, requiring manual untwisting. Examination of the intestinal tract after untwisting revealed no evidence of ischemia or necrosis. However, because a diverticulum was observed on the mesenteric side of the upper jejunum and considering the influence of secondary small bowel volvulus, partial small bowel resection was performed. The patient had a favorable postoperative course.

## Introduction

Chylous ascites (CA) are a form of ascite that leak from the intestinal lymphatic network and are caused by damage to the lymphatic vessels caused by blunt abdominal trauma, abdominal surgery, or compression/obstruction of the lymphatic vessels caused by malignant tumors or torsion [1]. Small bowel volvulus (SBV) is a rare emergency, accounting for 1% of all small bowel obstructions [2], while CA accumulation is also rare. Herein, we report a case of infected SBV associated with CA.

## Case report

A 93-year-old man presented to the emergency department with abdominal pain. He had tenderness in the upper abdomen, but no peritoneal irritation symptoms. His medical history included angina pectoris. He had undergone inguinal hernia surgery, but had no history of abdominal surgery.

**Figure 1:**
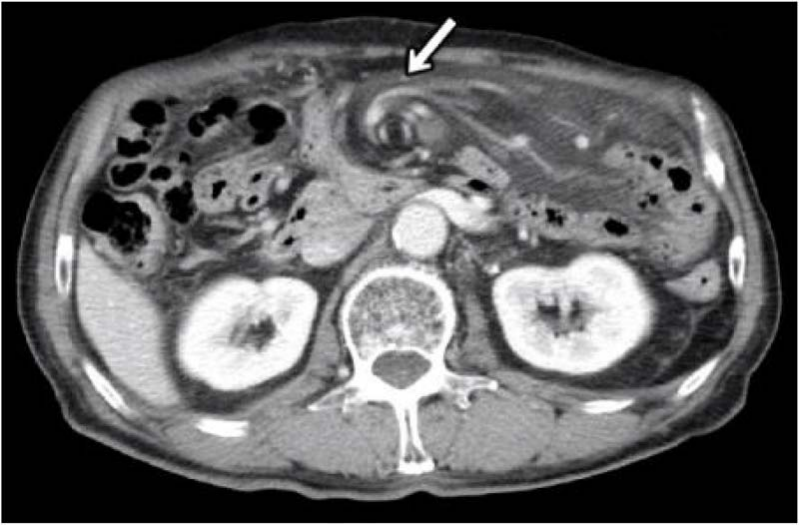
CT at initial diagnosis. CT revealed a whirl sign of the SMA and branches wrapped with the adjacent mesentery and small bowel loops, which are characteristic of SBV (allow).

Blood tests showed no elevated inflammatory findings (white blood cell count, 5.7 × 109/l, and C-reactive protein level, 0.15 mmol/l). Computed tomography (CT) of the abdomen and pelvis was performed (Fig. 1), revealing a small amount of ascites in the Douglas fossa, a whirl sign of the superior mesenteric artery (SMA), and branches wrapped with the adjacent mesentery and small bowel loops, all characteristic of SBV. Therefore, a diagnosis of SBV was made, emergency surgery was performed.

**Figure 2:**
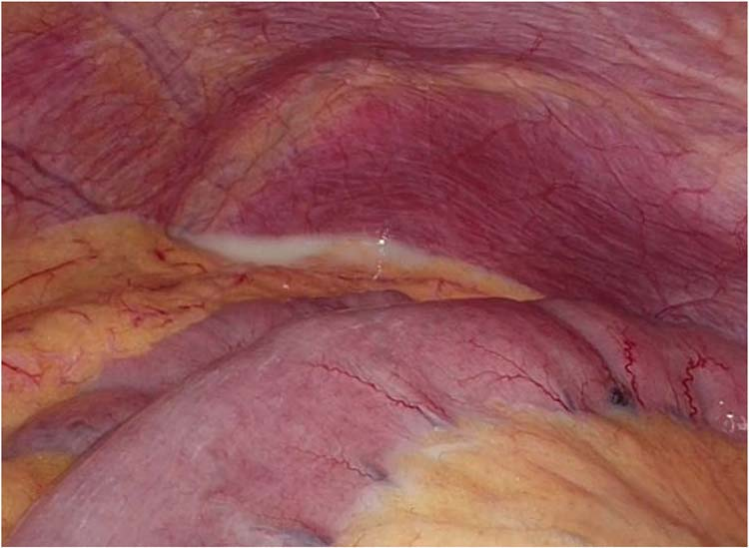
Intraoperative photograph showing CA in the abdominal cavity.

**Figure 3:**
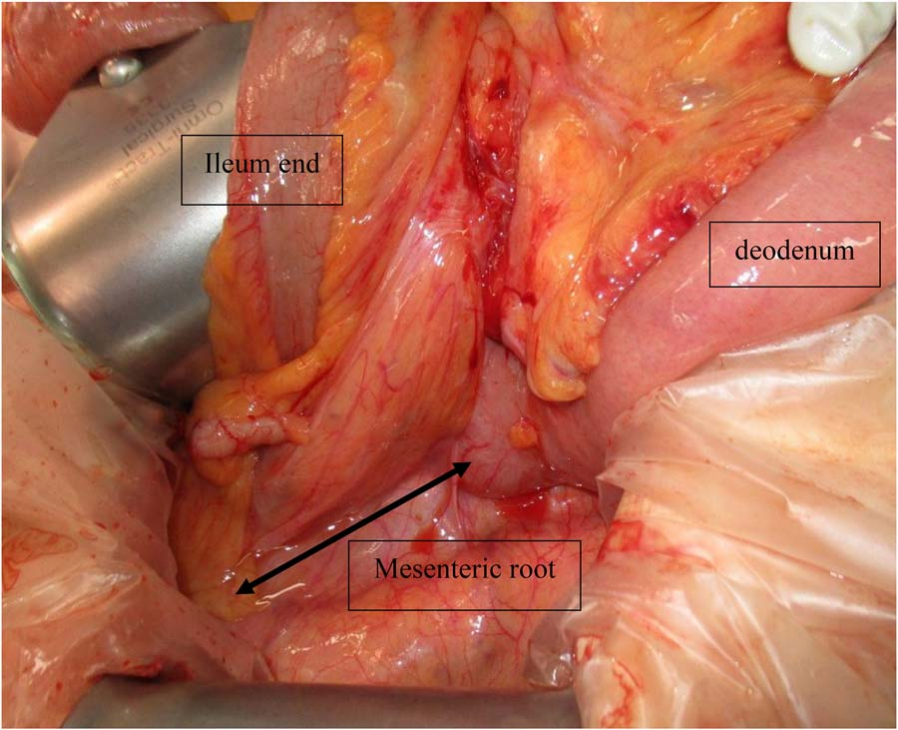
Intraoperative photograph showing that the entire small bowel was twisted 360° counterclockwise.

**Figure 4:**
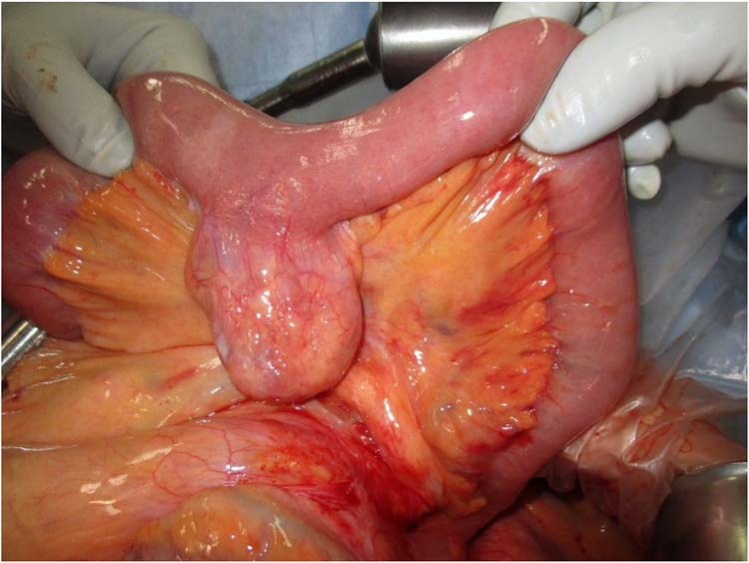
Intraoperative photograph showing a small palm-sized diverticulum on the mesenteric side of the upper jejunum.

Laparoscopic surgery was initiated. Initial laparoscopic examination revealed an edematous small intestine with mild congestion and milky ascites (Fig. 2). The triglyceride level in the ascitic fluid was 670 mg/dL. Although we attempted to release the torsion laparoscopically, it was difficult to manipulate with forceps because of the edematous intestinal tract and mesentery; therefore, the patient was transferred to laparotomy. The entire small bowel was observed to be twisted 360° counterclockwise, and was therefore manually untwisted (Fig. 3). The duodenum was not fixed to the retroperitoneum as a horizontal leg, but was free from the retroperitoneum on the right side of the vertebral body. We examined the intestinal tract after untwisting and found no evidence of ischemia or necrosis. However, because a small palm-sized diverticulum was observed on the mesenteric side of the upper jejunum, partial resection of the small bowel, including the diverticulum area, was performed, considering the influence of secondary SBV (Fig. 4). The patient had a good postoperative course, but developed angina during hospitalization and was transferred to the cardiology department on postoperative Day 23.

## Discussion

Cases of SBV can be classified as primary (without underlying disease or anatomic abnormality), or secondary (cases caused by a congenital or acquired predisposition). Congenital predispositions include abnormal bowel rotation and mesenteric fixation failure, whereas acquired predispositions include postoperative adhesions and tumors [3]. In the present case, the horizontal duodenal leg was inadequately fixed and the duodenum was free from the retroperitoneum on the right side of the vertebral body. The mesenteric root was shorter than normal, which makes the entire small intestine more prone to torsion. In addition to the congenital predisposition, this patient was also considered to have a secondary component due to a small bowel diverticulum; therefore, additional small bowel resection was performed.

CA is defined as milky ascites with a high degree of triglyceride (TG) exudation from the intestinal lymphatic network [4]. Prior reports have also indicated that a TG of 200 mg/dL or higher in ascites is useful for the diagnosis of CA. In the present case, the TG was as high as 670 mg/dL, which was diagnostic of CA [5]. The causes of CA include acquired factors such as malignancy, liver cirrhosis, and filariasis, as well as congenital factors such as congenital lymphatic malformations and intestinal rotation abnormalities [1]. The lymphatic channels converge toward the root of the mesentery and run along the SMA. We considered that the mechanism by which SBV causes CA involves the leakage of lymphatic fluid into the abdominal cavity by obstructing the lymphatic vessels due to low pressure [6].

Patients with SBV infection may present with abdominal pain, nausea, vomiting, bloating, and bowel obstruction [7]. However, these symptoms do not all co-occur in all patient. Abdominal contrast CT is useful in the diagnosis of SBV, and the whirl sign, in which the intestinal tract is spiraled around the mesenteric vessels, is considered characteristic [7].

SBV is a serious disease with a mortality rate of 10–35%, and early diagnosis and surgery are necessary to avoid intestinal necrosis and perforation [3, 7]. Trial laparotomy should be performed to determine the course of treatment [8]. Bowel resection is necessary for necrotic intestinal tracts; however, it has been reported that strangulated ileus with CA often preserves arteriovenous blood flow in the strangulated digestive tract, and likely does not require bowel resection [8]. In the present case, a palm-sized diverticulum was identified in the small intestine. As such, small bowel resection was performed; however, necrosis of the intestinal tract was not observed. A PubMed search revealed no cases of SBV infection with CA requiring bowel resection.

Herein we report a rare case of SBV exhibiting CA. No previously reported cases of SBV due to congenital predisposition, or secondary cases, were found during a literature search.

## References

[1] Murugan K , Spence RA. Chylous peritonitis with small bowel obstruction. Ulster Med J 2008;77:132–3. 18711621 PMC2516422

[2] Coe TM , Chang DC, Sicklick JK. Small bowel volvulus in the adult populace of the United States: results from a population-based study. Am J Surg 2015;210:201–210.e2. 10.1016/j.amjsurg.2014.12.048. 26002189 PMC4475430

[3] Roggo A , Ottinger LW. Acute small bowel volvulus in adults. A sporadic form of strangulating intestinal obstruction. Ann Surg 1992;216:135–41. 10.1097/00000658-199208000-00003. 1503517 PMC1242584

[4] Riza Altiparmak M , Avsar S, Yanik S. Chylous ascites and chylothrax due to constricitive pericarditis in a patient undergoing haemodialysis. Neth J Med 2004;62:59–61. 15127833

[5] Cárdenas A , Chopra S. Chylous ascites. Am J Gastroenterol 2002;97:1896–900. 10.1016/S0002-9270(02)04268-5. 12190151

[6] Pai A , Park JJ, Marecik SJ, Prasad LM. Midgut volvulus presenting with acute chylous peritonitis. Clin Case Rep 2014;2:159–61. 10.1002/ccr3.88. 25356277 PMC4184655

[7] Klein J , Baxstrom K, Donnelly S., et al. A fatal twist: volvulus of the small intestine in a 46-year-old woman. Case Rep Med 2015;2015:391093. 26612989 10.1155/2015/391093 PMC4647019

[8] Akama Y , Shimizu T, Fujita I., et al. Chylous ascites associated with intestinal obstruction from volvulus due to Petersen’s hernia: report of a case. Surg Case Rep 2016;2:77. 10.1186/s40792-016-0207-9. 27468960 PMC4965361

